# Protein tyrosine phosphatase non-receptor type 2 (PTPN2) gene polymorphisms (rs2542151, rs7234029) in Egyptian Behçet’s disease patients: a preliminary report

**DOI:** 10.1007/s10067-024-07128-7

**Published:** 2024-09-25

**Authors:** Doaa H. S. Attia, Marwa Alkaffas, Mervat Eissa, Laila Rashed, Rasha A. M. Khattab, Radwa Elzanaty, Rabab A. Khattab, Lamees A. Samy

**Affiliations:** 1 Rheumatology and Rehabilitation Department, Faculty of Medicine, Cairo University Hospitals, Saray El Manial Street, El Manial, Cairo, 11956 Egypt; 2 Medical Biochemistry and Molecular Biology Department, Faculty of Medicine, Cairo University, Saray El Manial Street, El Manial, Cairo, 11956 Egypt; 3 Clinical and Chemical Pathology Department, Faculty of Medicine, Beni-Suef University, Beni-Suef, Egypt; 4 Ophthalmology Department, Faculty of Medicine, Cairo University, Saray El Manial Street, El Manial, Cairo, 11956 Egypt; 5 Ophthalmology Department, Helwan University Student’s Hospital, Cairo, Egypt; 6 Clinical and Chemical Pathology Department, Faculty of Medicine, Cairo University Hospitals, Saray El Manial Street, El Manial, Cairo, 11956 Egypt

**Keywords:** Behçet’s disease, Polymorphisms, Protein tyrosine phosphatase non-receptor type 2, *PTPN2*, SNPs

## Abstract

Single nucleotide polymorphisms (SNPs) of the protein tyrosine phosphatase non-receptor type 2 (*PTPN2*) gene have been documented to be linked with several autoimmune disorders including Behçet’s disease (BD).* PTPN2* SNPs rs2542151 and rs7234029 have been assessed using real-time PCR in 96 BD patients and 50 controls matched by age and gender. Patients were categorized into groups according to the disease phenotypes and severity. A total of 94.8% of patients were males. The patients’ mean age at onset was 26.1 ± 8 years. The median (IQR) disease duration was 8.5(4–13) years. No difference was observed between the patients and controls concerning the frequency of the two SNPs’ different genotypes, models, and alleles. Moreover, neither disease phenotypes nor severity were associated with rs2542151 or rs7234029 SNPs.* PTPN2* rs2542151 and rs7234029 SNPs do not seem to have associations with BD occurrence, phenotypes, or severity in the Egyptian patients.

**Table:** 

**Key Points**
• *PTPN2 rs2542151 and rs7234029 SNPs do not seem to have associations with BD occurrence, phenotypes, or severity in the Egyptian patients.* • *Further studies involving a larger sample size with variable clinical diversity are recommended to verify the results.*

**Supplementary Information:**

The online version contains supplementary material available at 10.1007/s10067-024-07128-7.

## Introduction

Behçet’s disease (BD) is an inflammatory disease characterized by a relapsing course. It can potentially impact all organ systems; however, it is most likely to affect the vascular, neurological, mucocutaneous, and articular systems. Gender differences have been described globally. Males exhibit a higher prevalence of the disease in the Mediterranean region, whereas females are disproportionately afflicted in the Far East [1]. It is hypothesized that it incorporates characteristics of autoimmunity and autoinflammation. The exact mechanism by which BD develops is unknown. Although the disease risk factors remain unknown, genetic and environmental influences appear to be significant [2].

Patients with BD express different disease phenotypes in isolation or combination. Factors favoring the expression of a particular phenotype are not fully understood; however, a genetic contribution was suggested [3]. Identifying the environmental and genetic risk factors could help in understanding the disease pathogenesis and the phenotype-genotype interactions with the subsequent development of effective therapeutic agents.

Human leukocyte antigen (HLA)-B51 was the first studied gene in BD. It was thought to have the most potent genetic predisposition to the disease. However, it contributes to less than 20% of the genetic predisposition to the disease [4]. Many non-HLA genes were studied. Among the studied non-HLA genes is the protein tyrosine phosphatase non-receptor type 2 (*PTPN2*) gene [5, 6].

In cell cycle regulation and signal transduction, protein tyrosine phosphatases (PTPs) are crucial regulatory enzymes. Non-receptor-type PTPs (PTPN22, SHP-1, PTPN2, and PTP-PEST) are distinguished from receptor-type PTPs (CD45 and CD148) [6, 7].

Chromosome 18p11 contains the *PTPN2* gene. The term T cell protein tyrosine phosphatase (TC-PTP) is applied to its protein product [8]. It is a key regulatory factor of T-cell-mediated immune response through suppression of the Janus kinase/signal transducers and activators of transcription (JAK/STAT) signaling pathway following T cell and cytokines receptors activation [9–12]. PTPN2-deficient mice were found to express systemic inflammation and die shortly after birth [7, 13]. Moreover, it has been documented that *PTPN2* single nucleotide polymorphisms (SNPs) are linked to dysfunctional protein products with subsequent increment of proinflammatory cytokines production [14], upregulation of Th2 and Th17, downregulation of T_Reg_, and the development of autoimmunity [15].

Numerous studies addressed the role of *PTPN2* SNPs in several autoinflammatory/autoimmune diseases like inflammatory bowel disease (IBD) [14, 16–18], juvenile idiopathic arthritis [19], rheumatoid arthritis [20, 21], and type I diabetes mellitus [22–26].

Notably, Crohn’s disease (CD) and BD share common characteristics: (1) common age at onset, (2) common relapsing course, (3) major role of the innate immune system in the pathogenesis of both diseases, (4) contribution of normal flora of oral mucosa in BD pathogenesis and intestinal microbiota in CD pathogenesis, (5) gastrointestinal involvement that could be indistinguishable between the two diseases, (6) extraintestinal features that are common between both diseases, i.e., mucocutaneous, musculoskeletal, ocular, and vascular, (7) some patients with intestinal BD may be mismanaged as cases of CD while some CD patients could satisfy the classification criteria of BD [27–29].

While *PTPN2* SNPs are extensively studied in CD [14, 16–18], few studies addressed the association between *PTPN2* polymorphisms and BD in Chinese patients [5, 6]; and no data are available about other ethnicities. As most of the autoimmune rheumatic diseases show polygenic inheritance with shared susceptibility loci between different diseases [30, 31] and based on the aforementioned perspectives, this work aimed to study *PTPN2* SNPs (rs2542151 T → G and rs7234029 A → G) in Egyptian BD patients trying to find a relation between the two SNPs and disease occurrence, phenotypes and severity.

## Materials and methods

### Study design and data acquisition

Ninety-six adult patients were recruited from the Rheumatology and Rehabilitation Department and Outpatient Clinic of Kasr Al-Ainy Hospital, Cairo University. Fifty apparently healthy age- and sex-matches persons were included as control subjects. The 2006 International Study Group Criteria for BD were used for patients’ classification [32]. Patients with overlap syndromes were excluded.

All patients underwent thorough history taking, clinical examination, and laboratory testing. Ophthalmological assessment of patients was carried out in the Ophthalmology Department of Kasr Al-Ainy Hospital, Cairo University. The epidemiological features in addition to the clinical characteristics were collected from medical files using a standardized form (Table S1). Disease onset was defined as the time point of occurrence of the initial disease manifestation. Disease duration referred to the duration between the disease onset and enrollment in the study.

Patients were categorized into six phenotypes according to the involved organ system(s): mucocutaneous, ocular, neurological, peripheral vascular, musculoskeletal, and gastrointestinal phenotypes.

The disease severity score was calculated as suggested by Yosipovitch et al. (1995) [33] and modified by Krause et al. (2001) [34] categorizing the patients into one of three groups: mild, moderate, and severe. The presence of one of the severe manifestations is sufficient to categorize the patient as having a severe disease. The same applies to the moderately severe manifestations; otherwise, the patient is categorized as having a mild disease. Each mild, moderate, and severe item is given 1, 2, and 3 points, respectively, with the final score calculated as the sum of the total items reflecting the cumulative number of manifestations the patient has.

This research investigation received approval from the Research Ethics Committee of Faculty of Medicine, Cairo University (N-51–2023), and it conformed to the 1964 Helsinki Declaration and its subsequent revisions. Each subject provided an informed consent.

### SNP genotyping assay

Genotyping was performed using real-time polymerase chain reaction (PCR) with TaqMan allelic discrimination assay (Applied Biosystems, USA).

Peripheral blood samples in EDTA vacutainers were withdrawn from each patient to be stored below 20 °C until DNA extraction was performed. A 50 bp ladder (GeneRuler TM) was used to determine the size of the PCR fragments. Detection of *PTPN2* gene polymorphisms (rs2542151 and rs7234029) was conducted using allelic discrimination assay by Applied Biosystems (ABI) 7500 real-time PCR system (Foster City, CA, USA).

Thermal cycling was designed for 10 min for the initial denaturation cycle at 95 °C, then 50 cycles were conducted for 15 s at 95 °C, and finally, a 1-min cycle at 60 °C. The Lot. No. for the utilized Taq Man genotyping master mix from Applied Biosystems was 01245069. Genotyping was done in the PCR Unit of the Clinical and Chemical Pathology Department, Kasr Al-Ainy Hospital, Cairo University.

### Statistical methods

Data were coded and entered using the statistical package for the Social Sciences (SPSS) version 28 (IBM Corp., Armonk, NY, USA). Data were summarized using means and standard deviations for the normally distributed quantitative variables, medians and interquartile ranges for the non-normally distributed quantitative variables; and frequencies (number of cases) and relative frequencies (percentages) for categorical variables. Comparisons between numerical variables were done using the non-parametric Kruskal–Wallis test and Mann–Whitney test as appropriate [35]. For comparing categorical data, chi-square (χ2) test was performed. The exact test was used instead when the expected frequency was less than 5 [36]*.* Odds ratio (OR) with 95% confidence intervals were calculated. *P*-values less than 0.05 were considered statistically significant.

Power analysis of the current study was done on comparing genotypes between cases and controls, and between disease severity categories using our results as effect size. The chi-squared and Fisher exact tests for independent samples were chosen to perform the power analysis; the α-error level was fixed at 0.05. When the frequency table includes the value, “zero”, we added the category to the nearest one to perform the analysis. The percentage of each genotype was entered with the corresponding sample size in each group. Calculations were done using G*Power software version 3.1.9.6 for MS Windows, Franz Faul, Kiel University, Germany.

## Results

This study included 96 BD patients, 94.8% of whom were males. The patients’ mean age at onset was 26.1 ± 8 years. The median (IQR) disease duration was 8.5 (4–13) years.

The proportions of patients in the six phenotypes were 100%, 65.6%, 29.2%, 29.2%, 21.9%, and 4.2% for the mucocutaneous, ocular, peripheral vascular, neurological, musculoskeletal, and gastrointestinal phenotypes, respectively.

Clinical manifestations and disease severity of the patients are presented in Table 1. The median (IQR) severity score was 6 (5–8).

**Table 1:** Demographic features, clinical manifestations, and disease severity of the patient group

Characteristics (*N* = 96)		
Gender	Males, *N* (%)	91(94.8)
	Females, *N* (%)	5(5.2)
Constitutional manifestations, *N* (%)		22(22.9)
Mucocutaneous manifestations, *N* (%)		96(100)
Oral ulcers, *N* (%)		94(97.9)
Genital ulcers, *N* (%)		76(79.2)
Musculoskeletal involvement, *N* (%)		21(21.9)
Pulmonary vascular involvement, *N* (%)		7(7.3)
Ocular involvement, *N* (%)		63(65.6)
Neurological involvement, *N* (%)		28(29.2)
GIT involvement, *N* (%)		4(4.2)
Peripheral vascular disease, *N* (%)		28(29.2)
Deep venous thrombosis, *N* (%)		27(28.1)
Peripheral arterial disease, *N* (%)		7(7.3)
Great vein disease, *N* (%)		7(7.3)
Aortic aneurysm, *N* (%)		2(2.1)
Cardiac involvement, *N* (%)		1(1)
Disease severity	Mild, *N* (%)	5(5.2)
	Moderate, *N* (%)	11(11.5)
`	Severe, *N* (%)	80(83.3)
Age at onset, years, mean ± SD		26.1 ± 8
Disease duration, years, median (IQR)		8.5(4–13)
Disease severity score, median (IQR)		6(5–8)

*N* number, *GIT* gastrointestinal, *IQR* interquartile range

No significant difference was observed between the patients and controls regarding the frequency of the two SNPs’ different genotypes, genetic models, and alleles, as shown in Table 2.

**Table 2:** Comparisons between patients and controls regarding genotypes, models, and alleles frequencies of PTPN2 SNPs rs2542151 and rs7234029

SNP				Cases *N* (%) *N* = 96	Controls *N* (%) *N* = 50	*p* value	OR(95%CI)
**PTPN2 rs2542151**	Genotypes		GG	8(8.3)	2(4)	0.288	
			GT	31(32.3)	12(24)		
			TT	57(59.4)	36(72)		
	Models	Dominant	GG + GT	39(40.6)	14(28)	0.132	1.8(0.8–3.7)
			TT	57(59.4)	36(72)		
		Recessive	GG	8(8.3)	2(4)	0.495	2.2(0.4–10.7)
			GT + TT	88(91.7)	48(96)		
	Alleles		G	47(24.5)	16(16)	0.095	1.7(0.9–3.2)
			T	145(75.5)	84(84)		
**PTPN2 rs7234029**	Genotypes		GG	6(6.2)	3(6)	0.994	
			AG	28(29.2)	15(30)		
			AA	62(64.6)	32(64)		
	Models	Dominant	GG + AG	34(35.4)	18(36)	0.944	1(0.5–2)
			AA	62(64.6)	32(64)		
		Recessive	GG	6(6.2)	3(6)	> 0.999	1(0.3–4.4)
			AG + AA	90(93.8)	47(94)		
	Alleles		G	40(20.8)	21(21)	0.973	1(0.5–1.8)
			A	152(79.2)	79(79)		

*PTPN2* protein tyrosine phosphatase non-receptor type 2, *SNP* single nucleotide polymorphism, *N* number, *OR* odds ratio, *CI* confidence interval. Comparisons were done using the chi-square tests

Table 3 shows the association between *PTPN2* rs2542151 and the sex, age at disease onset, clinical characteristics, and disease severity at the genotypes, genetic models, and alleles levels. The comparisons revealed an association between the SNP and neurological involvement under the recessive model (*p* = 0.044, OR (95% CI)= 4.7 (1.0–21.3)).

**Table 3:** The associations between PTPN2 SNP rs2542151 genotypes, models, and alleles; and the demographic and clinical characteristics as well as disease severity of the patients group

Characteristics		Genotypes *N* = 96				Models								Alleles *N* = 192			
						Dominant *N* = 96				Recessive *N* = 96							
		GG (*N* = 8)	GT (*N* = 31)	TT (*N* = 57)	*p* value	GG + GT (*N* = 39)	TT (*N* = 57)	*p* value	OR(95%CI)	GG (*N* = 8)	GT + TT (*N* = 88)	*p* value	OR(95%CI)	G (*N* = 47)	T (*N* = 145)	*p* value	OR(95%CI)
Gender	Females, *N* (%)	0(0)	0(0)	5(8.8)	0.165	0(0)	5(8.8)	0.078		0(0)	5(5.7)	> 0.999	–-	0(0)	10(6.9)	0.123	–-
	Males, *N* (%)	8(100)	31(100)	52 (91.2)		39(100)	52(91.2)			8(100)	83(94.3)			47(100)	135(93.1)		
Constitutional manifestations, *N* (%)		2(25)	8(25.8)	12(21.1)	0.870	10(25.6)	12(21.1)	0.599	1.3(0.5–3.4)	2(25)	20(22.7)	> 0.999	1.1(0.2–6.1)	12(25.5)	32(22.1)	0.624	1.2(0.6–2.6)
Oral ulcers, *N* (%)		8(100)	29(93.5)	57(100)	0.117	37(94.9)	57(100)	0.162	–-	8(100)	86(97.7)	> 0.999	–-	45(95.7)	143(98.6)	0.252	0.3(0–2.3)
Genital ulcers, *N* (%)		7(87.5)	24(77.4)	45(78.9)	0.820	31(79.5)	45(78.9)	0.949	1(0.4–2.8)	7(87.5)	69(78.4)	> 0.999	1.9(0.2–16.6)	38(80.9)	114(78.6)	0.744	1.1(0.5–2.6)
Musculoskeletal involvement, *N* (%)		0(0)	7(22.6)	14(24.6)	0.288	7(17.9)	14(24.6)	0.441	0.7(0.2–1.9)	0(0)	21(23.9)	0.194		7(14.9)	35(24.1)	0.183	0.6(0.2–1.3)
Pulmonary vascular involvement, *N* (%)		1(12.5)	2(6.5)	4(7)	0.835	3(7.7)	4(7)	> 0.999	1.1(0.2–5.2)	1(12.5)	6(6.8)	0.467	2(0.2–18.6)	4(8.5)	10(6.9)	0.749	1.3(0.4–4.2)
Ocular involvement, *N* (%)		7(87.5)	18(58.1)	38(66.7)	0.285	25(64.1)	38(66.7)	0.795	0.9(0.4–2.1)	7(87.5)	56(63.6)	0.256	4(0.5–34)	32(68.1)	94(64.8)	0.683	1.2(0.6–2.3)
Neurological involvement, *N* (%)		5(62.5)	7(22.6)	16(28.1)	0.083	12(30.8)	16(28.1)	0.775	1.1(0.5–2.8)	5(62.5)	23(26.1)	***0.044***	4.7(1–21.3)	17(36.2)	39(26.9)	0.224	1.5(0.8–3.1)
GIT involvement, *N* (%)		0(0)	2(6.5)	2(3.5)	> 0.665	2(5.1)	2(3.5)	> 0.999	1.5(0.2–11)	0(0)	4(4.5)	> 0.999	–-	2(4.3)	6(4.1)	> 0.999	1(0.2–5.3)
Peripheral vascular disease, *N* (%)		2(25)	11(35.5)	15(26.3)	0.641	13(33.3)	15(26.3)	0.458	1.2(0.7–2)	2(25)	26(29.5)	> 0.999	0.8(0.2–4.2)	15(31.9)	41(28.3)	0.633	1.2(0.6–2.4)
Deep venous thrombosis, *N* (%)		2(25)	11(35.5)	14(24.6)	0.541	13(33.3)	14(24.6)	0.348	1.5(0.6–3.8)	2(25)	25(28.4)	> 0.999	0.8(0.2–4.4)	15(31.9)	39(26.9)	0.506	1.3(0.6–2.6)
Peripheral arterial disease, *N* (%)		0(0)	3(9.7)	4(7)	0.639	3(7.7)	4(7)	> 0.999	1.1(0.2–5.2)	0(0)	7(8)	> 0.999	–-	3(6.4)	11(7.6)	> 0.999	0.8(0.2–3.1)
Great vein disease, *N* (%)		0(0)	2(6.5)	5(8.8)	0.655	2(5.1)	5(8.8)	0.697	0.6(0.1–3.1)	0(0)	7(8)	> 0.999	–-	2(4.3)	12(8.3)	0.524	0.5(0.1–2.3)
Aortic aneurysm, *N* (%)		0(0)	1(3.2)	1(1.8)	0.819	1(2.6)	1(1.8)	> 0.999	1.5(0.1–24.3)	0(0)	2(2.3)	> 0.999	–-	1(2.1)	3(2.1)	> 0.999	1(0.1–10.1)
Cardiac involvement, *N* (%)		0(0)	0(0)	1(1.8)	0.708	0(0)	1(1.8)	> 0.999		0(0)	1(1.1)	> 0.999	––	0(0)	2(1.4)	> 0.999	–-
Disease severity	Mild, *N* (%)	0(0)	3(9.7)	2(3.5)	0.393	3(7.7)	2(3.5)	0.606	–-	0(0)	5(5.7)	0.418	–-	3(6.4)	7(4.8)	0.904	–-
	Moderate, *N* (%)	0(0)	5(16.1)	6(10.5)		5(12.8)	6(10.5)			0(0)	11(12.5)			5(10.6)	17(11.7)		
	Severe, *N* (%)	8(100)	23(74.2)	49(86)		31(79.5)	49(86)			8(100)	72(81.8)			39(83)	121(83.4)		
Age at onset, years, median (IQR)		22(20–26.5)	24(21–32)	26(21–30)	0.516	24(21–30)	26(21–30)	0.490	–-	22(20–26.5)	25(21–30)	0.268	–-	24(21–28)	25(21–30)	0.287	–-
Disease duration, years, median (IQR)		7(4–10.5)	8(5.5–12.5)	9(4–13)	0.625	8(5–12)	9(4–13)	0.934	–-	7(4–10.5)	9(4–13)	0.353	–-	8(5–12)	9(4–13)	0.624	–-
Disease severity score, median (IQR)		8(5–9.5)	6(5–7.5)	6(5–8)	0.509	6(5–8)	6(5–8)	0.862	–-	8(5–9.5)	6(5–8)	.257	–-	6(5–8)	6(5–8)	0.511	–-

*PTPN2* protein tyrosine phosphatase non-receptor type 2, *SNP* single nucleotide polymorphism, *N* number, *OR* odds ratio, *CI* confidence interval, *GIT* gastrointestinal. Comparisons were done using the chi-square tests for the qualitative variables and the Kruskal–Wallis or Mann–Whitney tests for the quantitative variables, as appropriate

There was no association between *PTPN2* rs7234029 and the sex, age at disease onset, clinical characteristics, and disease severity at the genotypes, genetic models, and alleles levels, as shown in Table 4.

**Table 4:** The association between PTPN2 SNP rs7234029 genotypes, models, and alleles; and the demographic and clinical characteristics as well as disease severity

Characteristics		Genotypes *N* = 96				Models								Alleles *N* = 192			
						Dominant *N* = 96				Recessive *N* = 96							
		GG (*N* = 6)	AG (*N* = 28)	AA (*N* = 62)	*p* value	GG + AG (*N* = 34)	AA (*N* = 62)	*p* value	OR(95%CI)	GG (*N* = 6)	AG + AA (*N* = 90)	*p* value	OR(95%CI)	G (*N* = 40)	A (*N* = 152)	*p* value	OR(95%CI)
Gender	Females, *N* (%)	1(16.7)	2(7.1)	2(3.2)	0.316	3(8.8)	2(3.2)	0.343	2.9(0.5–18.3)	1(16.7)	4(4.4)	0.281	4.3(0.4–46)	4(10)	6(3.9)	0.221	2.7(0.7–10.1)
	Males, *N* (%)	5(83.3)	26(92.9)	60(96.8)		31(91.2)	60(96.8)			5(83.3)	86(95.6)			36(90)	146(96.1)		
Constitutional manifestations, *N* (%)		2(33.3)	5(17.9)	15(24.2)	0.660	7(20.6)	15(24.2)	0.688	0.8(0.3–2.2)	2(33.3)	20(22.2)	0.618	1.8(0.3–10.3)	9(22.5)	35(23)	0.944	1(0.4–2.2)
Oral ulcers, *N* (%)		6(100)	28(100)	60(96.8)	0.571	34(100)	60(96.8)	0.538	–-	6(100)	88(97.8)	> 0.999	–-	40(100)	148(97.4)	0.582	–-
Genital ulcers, *N* (%)		4(66.7)	20(71.4)	52(83.9)	0.299	24(70.6)	52(83.9)	0.125	0.5(0.2–1.3)	4(66.7)	72(80)	0.601	0.5(0.1–2.9)	28(70)	124(81.6)	0.109	0.5(0.2–1.2)
Musculoskeletal involvement, *N* (%)		2(33.3)	9(32.1)	10(16.1)	0.184	11(32.4)	10(16.1)	0.066	2.5(0.9–6.7)	2(33.3)	19(21.1)	0.609	1.9(0.3–11)	13(32.5)	29(19.1)	0.608	2(0.9–4.4)
Pulmonary vascular involvement, *N* (%)		0(0)	3(10.7)	4(6.5)	0.600	3(8.8)	4(6.5)	0.695	1.4(0.3–6.7)	0(0)	7(7.8)	> 0.999	–-	3(7.5)	11(7.2)	> 0.999	1(0.3–3.9)
Ocular involvement, *N* (%)		3(50)	22(78.6)	38(61.3)	0.197	25(73.5)	38(61.3)	0.227	1.8(0.7–4.4)	3(50)	60(66.7)	0.411	0.5(0.1–2.6)	28(70)	98(64.5)	0.513	1.3(0.6–2.7)
Neurological involvement, *N* (%)		3(50)	7(25)	18(29)	0.473	10(29.4)	18(29)	0.969	1(0.4–2.6)	3(50)	25(27.8)	0.353	2.6(0.5–13.7)	13(32.5)	43(28.3)	0.602	1.2(0.6–2.6)
GIT involvement, *N* (%)		0(0)	0(0)	4(6.5)	0.318	0(0)	4(6.5)	0.294	–-	0(0)	4(4.4)	> 0.999	–-	0(0)	8(5.3)	0.208	–-
Peripheral vascular disease, *N* (%)		2(33.3)	7(25)	19(30.6)	0.839	9(26.5)	19(30.6)	0.667	0.8(0.3–2.1)	2(33.3)	26(28.9)	> 0.999	1.2(0.2–7.1)	11(27.5)	45(29.6)	0.794	0.9(0.4–2)
Deep venous thrombosis, *N* (%)		2(33.3)	7(25)	18(29)	0.886	9(26.5)	18(29)	0.789	0.9(0.3–2.3)	2(33.3)	25(27.8)	> 0.999	1.3(0.2–7.5)	11(27.5)	43(28.3)	0.921	1(0.4–2.1)
Peripheral arterial disease, *N* (%)		0(0)	1(3.6)	6(9.7)	0.457	1(2.9)	6(9.7)	0.415	0.3(0–2.5)	0(0)	7(7.8)	> 0.999	–-	1(2.5)	13(8.6)	0.308	0.3(0–2.2)
Great vein disease, *N* (%)		1(16.7)	3(10.7)	3(4.8)	0.403	4(11.8)	3(4.8)	0.240	2.6(0.6–12.5)	1(16.7)	6(6.7)	0373	2.8(0.3–28)	5(12.5)	9(5.9)	0.174	2.3(0.7–7.2)
Aortic aneurysm, *N* (%)		0(0)	1(3.6)	1(1.6)	0.779	1(2.9)	1(1.6)	> 0.999	1.8(0.1–30.5)	0(0)	2(2.2)	> 0.999	–-	1(2.5)	3(2)	> 0.999	1.3(0.1–12.6)
Cardiac involvement, *N* (%)		0(0)	1(3.6)	0(0)	0.293	1(2.9)	0(0)	0.354	__	0(0)	1(1.1)	> 0.999	–-	1(2.5)	1(0.7)	0.374	3.9(0.2–63.3)
Disease severity	Mild, *N* (%)	0(0)	1(3.6)	4(6.5)	0.470	1(2.9)	4(6.5)	0.603	–-	0(0)	5(5.6)	0.200	–––	1(2.5)	9(5.9)	0.303	–-
	Moderate, *N* (%)	2(33.3)	3(10.7)	6(9.7)		5(14.7)	6(9.7)			2(33.3)	9(10)			7(17.5)	15(9.9)		
	Severe, *N* (%)	4(66.7)	24(85.7)	52(83.9)		28(82.4)	52(83.9)			4(66.7)	76(84.4)			32(80)	128(84.2)		
Age at onset, years, median (IQR)		24(21–37)	25.5(23–32)	25(21–30)	0.861	25.5(22–32)	25(21–30)	0.589	–-	24(21–37)	25(21–30)	0.909	–-	25.5(21.5–32)	25(21–30)	0.617	–-
Disease duration, years, median (IQR)		7.5(3–11)	9(6–14.5)	8.5(4–13)	0.778	8.5(6–14)	8.5(4–13)	0.594	–-	7.5(3–11)	9(4–13)	0.802	–-	8(5–13.5)	9(4–13)	0.734	–-
Disease severity score, median (IQR)		7.5(6–9)	6(5–8.5)	6(5–8)	0.563	6(5–9)	6(5–8)	0.620	–-	7.5(6–9)	6(5–8)	0.287	–-	6(5–9)	6(5–8)	0.388	–-

*PTPN2* protein tyrosine phosphatase non-receptor type 2, *SNP* single nucleotide polymorphism, *N* number, *OR* odds ratio, *CI* confidence interval, *GIT* gastrointestinal. Comparisons were done using the chi-square tests for the qualitative variables and the Kruskal–Wallis or Mann–Whitney tests for the quantitative variables, as appropriate

Regarding the relation between *PTPN2* SNPs rs2542151 and rs7234029 and the different disease phenotypes, only four phenotypes were included in the statistical analysis: the musculoskeletal, ocular, peripheral vascular, and neurological phenotypes. The mucocutaneous phenotype was excluded as it was universal in all patients. Patients showing an overlap of two or more phenotypes, excluding the mucocutaneous one, were excluded as well. Finally, 46 patients were included in the analysis: 3 patients with the musculoskeletal phenotype, 28 patients with the ocular phenotype, 6 patients with the neurological phenotype, and 9 patients with the peripheral vascular phenotype. Each one has the mucocutaneous phenotype plus one of the other four phenotypes. The analysis revealed that neither rs2542151 nor rs7234029 was associated with the disease phenotypes (Table 5).

**Table 5:** The relations between PTPN2 SNPs rs2542151 and rs7234029, and the different disease phenotypes

SNP				Musculoskeletal (*N* = 3)	Ocular (*N* = 28)	Neurological (*N* = 6)	Peripheral vascular (*N* = 9)	*p* value
**PTPN2 rs2542151**	Genotypes (***N*** = 46)		GG	0(0)	2(7.1)	1(16.7)	0(0)	0.900
			GT	1(33.3)	7(25)	1(16.7)	3(33.3)	
			TT	2(66.7)	19(67.9)	4(66.7)	6(66.7)	
	Models	Dominant (***N*** = 46)	GG + GT	1(33.3)	9(32.1)	2(33.3)	3(33.3)	
			TT	2(66.7)	19(67.9)	4(66.7)	6(66.7)	> 0.999
		Recessive (***N*** = 46)	GG	0(0)	2(7.1)	1(16.7)	0(0)	
			GT + TT	3(100)	26(92.9)	5(83.3)	9(100)	0.600
	Alleles (***N*** = 92)		G	1(16.7)	11(19.6)	3(25)	3(16.7)	
			T	5(83.3)	45(80.4)	9(75)	15(83.3)	0.950
**PTPN2 rs7234029**	Genotypes (***N*** = 46)		GG	0(0)	0(0)	1(16.7)	1(11.1)	0.081
			AG	0(0)	12(42.9)	0(0)	1(11.1)	
			AA	3(100)	16(57.1)	5(83.3)	7(77.8)	
	Models	Dominant (***N*** = 46)	GG + AG	0(0)	12(42.9)	1(16.7)	2(22.2)	0.270
			AA	3(100)	16(57.1)	5(83.3)	7(77.8)	
		Recessive (***N*** = 46)	GG	0(0)	0(0)	1(16.7)	1(11.1)	0.205
			AG + AA	3(100)	28(100)	5(83.3)	8(88.9)	
	Alleles (***N*** = 92)		G	0(0)	12(21.4)	2(16.7)	3(16.7)	
			A	6(100)	44(78.6)	10(83.3)	15(83.3)	0.626

*PTPN2* protein tyrosine phosphatase non-receptor type 2, *SNP* single nucleotide polymorphism, *N* number. Comparisons were done using the chi-square tests

The study has a power of 81.6% and 90.3% for testing the association between the *PTPN2* SNPs rs2542151, and disease occurrence and severity category, respectively. On the other side, the corresponding power for the other SNP, rs7234029, was 20.6% and 44.4%, respectively.

## Discussion

Based on the previously reported association between *PTPN2* SNPs and different autoimmune diseases [14–26] and the scarcity of data about BD [5, 6], we investigated *PTPN2* SNPs (rs2542151 T → G and rs7234029 A → G) in Egyptian patients with BD trying to find an association with disease susceptibility, phenotypic expression, and severity.

Regarding the prevalence of the two SNPs’ various genotypes, models, and alleles, we found no distinction between BD patients and controls. Analyzing the association between the two SNPs and the different disease phenotypes and severity revealed an association between rs2542151 SNP and neurological involvement under the recessive model; however, with a marginal *p*-value and a wide 95% CI (*p* = 0.044, OR (95% CI)= 4.7 (1.0–21.3)).

Our results agree with Wu and colleagues who reported a weak association between *PTPN2* rs2542151 GT genotype and BD risk (*p* = 0.024) in their Chinese Han population. This association was noted with the initial statistical analysis; however, it did not withstand after Bonferroni correction. On the contrary, the same SNP was associated with gastrointestinal involvement when patients were compared with the controls (*p* = 0.032). Conversely, rs7234029 SNP did not show any association [5].

In disagreement with the study mentioned above and ours, Zhang et al. reported an increased risk of ocular BD development with the *PTPN2* SNP rs7234029 AG genotype (*p* = 3.33 × 10^−8^, OR = 1.549) while the risk was lower with the AA (*p* = 1.10 × 10^−3^, OR = 0.772) and GG (*p* = 1.94 × 10^−5^, OR = 0.466) genotypes in their Han Chinese cohort with ocular BD. Moreover, these associations were observed with the co-dominant, dominant, recessive, and overdominant models. The previous associations were reported in men but not in women. Regarding the extra-ocular disease manifestations, the aforementioned SNP exhibited a significant correlation with genital ulcers, cutaneous lesions, and a positive pathergy test. Nevertheless, the authors acknowledged that this result might be biased by the predominance of males in their cohort [6].

On the contrary, *PTPN2* rs2542151 SNP did not show any association. Interestingly, the authors found significantly decreased *PTPN2* mRNA expression with the AA and AG genotypes compared with the GG genotype (*p* = 0.014 and 0.019, respectively). Moreover, TNF-α production was significantly lower with the GG genotype compared with the AA or AG genotypes [6].

The different ethnicities, population characteristics, and sample size as well as the influence of environmental and other genetic factors could explain the discrepancy between our study and others.

Being the first study of PTPN2 in Egyptian BD patients, this preliminary study was commenced using a representative sample of patients and controls: 96 patients and 50 control subjects. After getting the results, power analyses of the study were performed. The study has a power of 81.6% and 90.3% for testing the association between the *PTPN2* SNP rs2542151, and disease occurrence and severity category, respectively. On the other side, the corresponding power for the other SNP, rs7234029, was 20.6% and 44.4%, respectively, probably due to the weak association between this SNP and the disease. Moreover, the population of this study showed limited heterogeneity: 94.8% of the study cohort were males; 80% of patients had a severe disease; and few patients had the gastrointestinal phenotype, reflecting the disease characteristics in Egypt [37]. Hence, further studies, particularly involving rs2542151 SNP as a potential genetic determinant of the disease, are recommended using a larger sample size and a broader disease spectrum to confirm the study results.

## Conclusion

*PTPN2* rs2542151 and rs7234029 SNPs do not seem to have associations with BD development, phenotypic expression, or severity in Egyptian patients. Further studies involving a larger sample size with variable clinical diversity are recommended to verify the results.

## Supplementary Information

Below is the link to the electronic supplementary material.

Supplementary file1 (DOCX 13.7 KB)

## Data Availability

Data of this work are available upon request.
