# Supplementary material for: Protein tyrosine phosphatase non-receptor type 2 (PTPN2) gene polymorphisms (rs2542151, rs7234029) in Egyptian Behçet’s disease patients: a preliminary report
Source: Clin Rheumatol. 2024 Sep 25;43(11):3439–48. doi: 10.1007/s10067-024-07128-7 (PMC11489223; doi:10.1007/s10067-024-07128-7)
Supplement: Supplementary file 1 — Supplementary file1 (DOCX 13.7 KB) [file 10067_2024_7128_MOESM1_ESM.docx]

| **Table S1: Collected Items** |
| --- |
| - Code - Group - Gender - Age (years) - Age at onset (years) - Disease duration (years) - Constitutional manifestations - Oral ulcers - Genital ulcers - Other skin manifestations (specify) - Mucocutaneous phenotype - Arthritis - Arthralgia - Musculoskeletal phenotype - Superficial thrombophlebitis - Deep venous thrombosis - Great vein disease - Peripheral arterial disease - Aortic disease - Pulmonary artery disease - Peripheral vascular phenotype - Cardiac disease - Anterior segment eye disease - Posterior segment eye disease - Ocular phenotype - Neurological phenotype - GIT bleeding - GIT perforation - Gastrointestinal phenotype - Other disease manifestations (specify) - Numeric Severity Score - Categorical severity: Mild, moderate or severe - PTPN2 rs2542151 genotype - PTPN2 rs7234029 genotype |
